# Revisiting the Asian Buffalo Leech (*Hirudinaria manillensis*) Genome: Focus on Antithrombotic Genes and Their Corresponding Proteins

**DOI:** 10.3390/genes14112068

**Published:** 2023-11-12

**Authors:** Zichao Liu, Fang Zhao, Zuhao Huang, Qingmei Hu, Renyuan Meng, Yiquan Lin, Jianxia Qi, Gonghua Lin

**Affiliations:** 1 Engineering Research Center for Exploitation and Utilization of Leech Resources in Universities of Yunnan Province, School of Agriculture and Life Sciences, Kunming University, Kunming 650214, China; abclzc@aliyun.com (Z.L.); hwxhqm476@163.com (Q.H.); 17854335708@163.com (R.M.); 2 School of Life Sciences, Jinggangshan University, Ji’an 343009, China; zf_lgh@163.com (F.Z.); hzhow@163.com (Z.H.); lin523231314999@sina.com (Y.L.); 3 Nujiang Management Bureau of Gaoligongshan National Nature Reserve, Nujiang 673199, China; 18288294601@163.com

**Keywords:** leech, genome, antithrombotic protein, gene family, hirudin, anticoagulant activity

## Abstract

Leeches are well-known annelids due to their obligate blood-feeding habits. Some leech species secrete various biologically active substances which have important medical and pharmaceutical value in antithrombotic treatments. In this study, we provided a high-quality genome of the Asian buffalo leech (*Hirudinaria manillensis*), based on which we performed a systematic identification of potential antithrombotic genes and their corresponding proteins. Combining automatic and manual prediction, we identified 21 antithrombotic gene families including fourteen coagulation inhibitors, three platelet aggregation inhibitors, three fibrinolysis enhancers, and one tissue penetration enhancer. A total of 72 antithrombotic genes, including two pseudogenes, were identified, including most of their corresponding proteins forming three or more disulfide bonds. Three protein families (LDTI, antistasin, and granulin) had internal tandem repeats containing 6, 10, and 12 conserved cysteines, respectively. We also measured the anticoagulant activities of the five identified hirudins (hirudin_Hman1 ~ hirudin_Hman5). The results showed that three (hirudin_Hman1, hirudin_Hman2, and hirudin_Hman5), but not the remaining two, exhibited anticoagulant activities. Our study provides the most comprehensive collection of antithrombotic biomacromolecules from a leech to date. These results will greatly facilitate the research and application of leech derivatives for medical and pharmaceutical purposes in the treatment of thrombotic diseases.

## 1. Introduction

Thrombosis refers to a variety of disorders characterized by aberrant blood clots in the blood that restrict or occlude the lumen of blood vessels, resulting in the ischemia and infarction of organs and malfunction [1]. Thrombophilia is the most important component of cardiovascular diseases. According to the World Health Organization, cardiovascular diseases such as ischemic heart disease and stroke directly or indirectly related to thrombophilia claim more than 15 million lives each year worldwide, accounting for 27% of the world’s total deaths [2]. Pharmaceutical industries have developed a wide range of anticoagulation, anti-platelet aggregation, and fibrinolysis drugs as thrombosis prevention and therapeutic alternatives. Anticoagulants include warfarin, heparin sodium, argatroban, and rivaroxaban; anti-platelet aggregation drugs include aspirin, clopidogrel, and abciximab; while fibrinolytic drugs include streptokinase, alteplase, and ralteplase [3].

Although antithrombotic drugs have slowed the occurrence of thrombotic events in patients, their success in reducing the lethality of thrombotic disease is limited. The primary reason for this failure is that these drugs generally rely on a single target of action, and it is difficult to account for individual differences in dosing, which ultimately leads to frequent drug resistance, internal bleeding, liver and kidney damage, and other serious side effects that put patients’ lives at risk. For instance, warfarin, an anticoagulant, may lead to adverse events, including cerebral microhemorrhage, hemorrhagic stroke, subarachnoid hemorrhage, and leukocyte rupture vasculitis [4,5]. The use of the antiplatelet aggregation drug clopidogrel carries the risk of both drug resistance and increased all-cause mortality [6]. On the other hand, the fibrinolytic drugs streptokinase and ralteplase have the potential to cause gastrointestinal bleeding [7]. Thus, the development of safe and effective multi-target drugs with minimal side effects is a crucial direction for the treatment of thrombotic diseases.

Leeches (Hirudinea, Annelida) are well-known annelids due to their obligate blood-feeding habits and are found on every continent except Antarctica [8]. Over 800 species of leeches have been identified [9], and some of them are classified as medicinal leeches [10]. Numerous leeches feed on mammalian blood. To ensure a smooth satiation within a few minutes, they secrete various biologically active substances, including anticoagulants, thrombolytics, and anti-inflammatories, aimed at countering the host’s physiological hemostatic process [11]. If used correctly, these bioactive substances can have significant value in medicine and medicinal treatments [12]. The European medical leech (*Hirudo medicinalis*) has been a prevalent tool in the treatment of various ailments in European countries since the 17th century. These conditions include, but are not limited to, osteoarthritis [13], chronic pain [14], cutaneous leishmaniasis [15], and postoperative thrombosis prevention [16,17]. The use of dried leeches, such as *Hirudo nipponia*, is a common practice in traditional Chinese medicine for treating various diseases, particularly those related to thrombosis, including stroke and coronary heart disease. These leeches are often administered orally and have been widely studied by researchers [12,18,19].

There are many antithrombotic active components in leeches [11,20], but the one that has received the most attention is hirudin. Hirudin has a molecular weight of approximately 7000 Daltons and comprises approximately 65 amino acid residues [21,22]. Hirudin is the most potent natural thrombin inhibitor discovered to date, as it binds to thrombin and forms an exceedingly stable non-covalent complex [23,24]. Compared to anticoagulants such as warfarin and heparin, hirudin causes fewer bleeding side effects [25]. In combination with hirudin, leeches possess numerous antithrombotic bioactive compounds within their bodies and secreted saliva. For instance, bdellin, like hirudin, lengthened activated partial thromboplastin time and exerted anticoagulant capability [26]. Destabilase [27], leech carboxypeptidase inhibitor [28], and hyaluronidase [29] were believed to have thrombolytic properties. Saratin and leech antiplatelet protein (LAPP) were reported as having anti-platelet aggregation function [30]. Furthermore, antistasin and eglin C have been observed to have analgesic and anti-inflammatory properties and may indirectly affect thrombophilia [31,32]. To our knowledge, over twenty leech-derived antithrombotic active ingredients, involving over fifty gene loci, have been isolated [9,33,34].

There are significant variations in the genomes of different leech species which lead to considerable disparities in the composition, function, and evolution of genes related to thrombosis prevention [35]. Notably, previous studies have shown that the architecture, functional activity, and copy number of genes encoding hirudin can be completely different among diverse leech species [36]. These variations present a challenge to genetic analysis, yet also offer limitless potential for the functional application of these active molecules.

The Asian buffalo leech (*Hirudinaria manillensis* (Lesson, 1842), homotypic synonym: *Poecilobdella manillensis*) is classified under the family Hirudinidae, just like the European medicinal leech *H. medicinalis*. However, it is highly specialized in parasitizing mammals, as opposed to its counterpart. Sawyer [8] noted that this species is commonly found in natural water bodies located in South East Asian countries such as China, Indonesia, Myanmar, the Philippines, Thailand, and Vietnam [37,38]. This species is adapted to feeding on mammalian blood and possesses strong jaws specialized for piercing the tough hides of water buffaloes and elephants [39]. Additionally, the Asian buffalo leech is utilized as a medical and medicinal tool [40]. Adult specimens typically measure 130–140 mm in length and 10–13 mm in width. In laboratory conditions, a fully fed Asian buffalo leech can weigh over 50 g, which is significantly heavier than the average medical leech (*Hirudo* spp.). Furthermore, it has been shown that the antithrombotic properties of the Asian buffalo leech are superior to those of commonly used *Hirudo* species, including *H. medicinalis* and *H. nipponia* [41]. As a result, there is a great deal of interest in the functional genes of the Asian buffalo leech and their potential applications in medicine.

The rapid advancement of the high-throughput sequencing technology means that functional gene research on Asian buffalo leech has reached the level of genomics. Guan et al. [42] reported a draft genome using PacBio RSII sequencing platforms. They created an assembly (GenBank accession: GCA_015345955.1) of 151.8 Mb with a total of 467 scaffold and an N50 of 2.28 Mb. More recently, Zheng et al. [35] published another genome utilizing the Nanopore PromethION platform. Using chromosome conformation capture (Hi-C) technology, a chromosome-level genome was obtained. It is worth noting that due to the complex structure of the antithrombotic genes, some genes as well as the corresponding proteins were not included in the genome annotation process. Using hirudins as an example, Guan et al. [42] and Zheng et al. [35] identified only one and three hirudins, respectively. However, our investigation revealed the presence of at least five hirudin encoded genes in the two genomes (details below). Given their considerable application potential, high complexity, and intraspecific variability, it is essential to determine the composition and sequence variation of these proteins. Here, we present the chromosome-level genome of an additional sample of the Asian buffalo leech and systematically analyze the proteins related to antithrombotic activities in this species.

## 2. Materials and Methods

### 2.1. The DNA and RNA Sequencing

*H. manillensis* specimens were collected from Fangchenggang, Guangxi, China (coordinates: E 107°51′1.47″, N 21°41′13.65″). Following the removal of their digestive tracts, genomic DNA was collected from fresh tissue using the DNeasy Blood and Tissue Kit (Qiagen, Germany). The quality and integrity of the DNA samples were evaluated through agarose gel electrophoresis, NanoDrop spectrophotometry (NanoDrop Technologies, Wilmington, DE, USA) and Qubit fluorometry (Thermo Fisher Scientific, Waltham, MA, USA). Subsequently, the genomic DNA, which met the standards of quality and quantity, was utilized for building the PacBio and Illumina libraries.

HiFi SMRTbell libraries were prepared with the SMRTbell Express Template Prep Kit 2.0 (PacBio, Menlo Park, CA, USA). The genome DNA was fragmented to 15–18 kb using a g-TUBE (Covaris, Woburn, MA, USA), and damage and fragment ends were repaired with reagents provided in the Template Prep Kit. The repaired ends were then ligated with SMRTbell hairpin adapters, and AMPure PB beads (PacBio, Menlo Park, CA, USA) were employed to concentrate and purify the library. To acquire SMRTbell libraries with large inserts, templates larger than 15 kb were selected using the BluePippin system (SageScience, Beverly, MA, USA). The sequencing procedure was carried out by BioMarker (Beijing, China) utilizing the PacBio Sequel II platform. Thereafter, high-precision HiFi reads were generated using the CCS software (https://github.com/PacificBiosciences/ccs, accessed on 19 April 2022) with default settings.

Hi-C was performed using the following protocol: the leech tissues were fixed in 1% formaldehyde solution. Nuclear chromatin was obtained from the fixed tissue and digested using HindIII (New England Biolabs [NEB], Ipswich, MA, USA). The overhangs were blunted with bio-14-dCTP (Invitrogen, Carlsbad, CA, USA) and Klenow enzyme (NEB). After dilution and religation using T4 DNA ligase (NEB), the genomic DNA was extracted and sheared to 350–500 bp with a Bioruptor (Diagenode, Seraing, Belgium). The biotin-labeled DNA fragments were enriched with streptavidin beads (Invitrogen) and were sequenced using the Illumina HiSeq 2000 sequencing platform (Illumina Inc., San Diego, CA, USA) with both directions of 150 bp reads. We also generated ~100× short reads (called Survey reads) for polishing the genome assembly. DNA libraries with ~350 bp insertions were constructed and were then sequenced with both directions of 150 bp reads using the Illumina HiSeq 2000 sequencing platform. Quality control for raw reads of Hi-C and Survey was performed using fastp 0.20.0 with default settings and parameters [25].

For RNASeq, total RNA was extracted from head tissue using TRIzol reagent (Invitrogen) and purified using the RNeasy Mini Kit (Qiagen, Chatsworth, CA, USA). Oligo(dT)-loaded magnetic beads were used to purify poly(A) mRNA from total RNA. The mRNA was then fragmented into small pieces (300~500 bp) at 94 °C for exactly 5 min. The cleaved RNA fragments were reverse-transcribed into first-strand cDNA using SuperScript II reverse transcriptase and random primers, and second-strand cDNA was generated using GEX second-strand buffer, DNA polymerase, RNase H, and dNTPs. These cDNA fragments were subjected to end repair and 3′ adenylation. Paired-end adapters were ligated to the 3′-adenylated cDNA fragments. cDNA fragments of ~300 bp were selected and enriched through 15 cycles of PCR amplification using Phusion DNA Polymerase. Finally, the constructed cDNA libraries were sequenced bidirectionally (150 bp in each direction) using the Illumina HiSeq 2000 sequencing platform. Quality control of raw RNASeq reads was also performed using fastp 0.20.0 with default settings and parameters.

### 2.2. Genome Assembling and Gene Prediction

The genome was assembled using PacBio HiFi reads through NextDenovo V2.5.0 (https://github.com/Nextomics/NextDenovo, accessed on 3 November 2021). The initial HiFi assembly contigs underwent polishing with Illumina PCR-free data utilizing NextPolish v1.4.0 [43] with recommended settings. For genome assembly at the chromosomal level, Hi-C sequencing reads were utilized with combined usage of samtools v1.3.1 [44], chromap v0.2.5 [45], Juicer v1.6.2 [46], and YaHS v1.1a [47] programs. The generated files (.hic and .assembly) by YaHS were imported into Juicebox v1.11.08 [48] for visualizing Hi-C maps and undergoing manual optimization. Juicer v1.6.2 was employed to generate the final pseudo-chromosome assemblies. In addition, we used the Survey reads to perform mitochondrial genome assembly using GetOrganelle v1.7.7.0 [49] with default settings.

BUSCO (Benchmarking Universal Single-Copy Orthologs) v.4.1.4 [50] with the eukaryota_odb10 database was used to assess the completeness of the genome assembly. We also used Merqury [51] to estimate the quality of the assembly. Both de novo and homology approaches were used to identify repetitive sequences in the leech genomes. First, RepeatModeler v2.0.3 [52] was used to construct the de novo libraries, which were then merged with the Annelida repeat sequences from the RepBase database v20181026 [53]. Finally, RepeatMasker v4.1.2-pl [52] was used to search for repeat sequences, and the repeat-masked genomes were used for gene prediction.

Both ab initio and RNASeq-based prediction strategies were used to predict protein-coding genes in the masked genomes. For the ab initio prediction strategy, GlimmerHMM v3.0.4 [54] and SNAP v2006-07-28 [55] were used with default settings to predict genes in the genome sequences. As for the RNASeq-based prediction strategy, three approaches were applied: (1) for the PASA approach, Trinity v2.9.0 [56] was used to assemble RNASeq reads to generate unigenes, and gene information was predicted using PASA v 2.5.2 [57]; (2) for the Stringtie approach, HISAT v2.147 [58] was used to align RNASeq reads to the genome, and gene information was predicted using StringTie v1.3.4c48 [59]; (3) for the BRAKER approach, STAR v2.7.9a [60] was used to align the RNASeq reads to the genome, and gene information was predicted using BRAKER v2.1.6 [61]. Finally, the General Feature Format (GFF) files generated from the above approaches were combined and integrated using EVidenceModeler v1.1.1 [62] to generate high-confidence gene sets (evm.gff). The coding sequences (CDS) and the corresponding protein sequences of all protein-coding genes were extracted using gffread v0.12.7 [63]. All protein sequences were functionally annotated using the NCBI NR, Uniprot TrEMBL, eggNOG [64], and Pfam [65] databases using BlastP with an E-value ≤ 1e-5.

### 2.3. Identification of Antithrombotic Genes

To the best of our knowledge, we collected all the available literature on leech antithrombotic genes and/or proteins. Sequence information was obtained directly from the literature or retrieved from genetic databases (GenBank, Uniprot, etc.) according to the accession number in the literature. All coding sequences were translated into protein sequences using MEGA software. These reported leech antithrombotic proteins (RAPs) were used as query sequences to identify homologous proteins in the *H. manillensis* genome. It should be mentioned that the above automated gene prediction approaches varied in their predictive sensitivity to antithrombotic genes. Even the relatively best BRAKER approach could predict only three out of five hirudins (see below). Fortunately, there are many RNASeq reads of *H. manillensis* (accession SRR15881191~SRR15881251) in the sequence reads archive of GenBank, which greatly facilitated our manual prediction on antithrombotic genes.

To obtain as many antithrombotic genes as possible, we used a so-called BRAKER-plus strategy to improve the prediction of potential antithrombotic genes. First, we assembled the RNA-Seq data sequenced in this study and those from GenBank using Trinity. The generated unigenes were used for CDS prediction using GeneMarkS-T v5.1 [66]. Second, we used the RAPs as queries to blast and extract all candidate antithrombotic genes from the RNASeq-derived CDS. All of these candidate genes were then mapped to the *H. manillensis* genome using the est2genome model in Exonerate v2.2.0 [67]. The highly matched regions were retained and manually checked to remove redundant or low-quality information. Third, the GFF files from the manual prediction and the above BRAKER prediction were merged using AGAT v1.2.0 [68]. After cleaning duplicated features between manual prediction and BRAKER prediction, we obtained a final version of the GFF file called BRAKER-plus.gff, which had updated information on antithrombotic genes.

### 2.4. Variation Analysis of Antithrombotic Proteins

The CDS of all antithrombotic genes were translated into protein sequences using Seqkit v0.10.2 [69]. The signal peptide region of each protein was predicted using the online tool SinalP (https://dtu.biolib.com/SignalP-6, accessed on 5 September 2023). For each gene family, the proteins predicted in this study and the corresponding archetypal protein were combined and aligned using Clustal W in MEGA v11.0.13 [70]. The protein families with extremely high mutation rates were manually verified. The pairwise sequence similarity of the proteins was calculated using the water program from EMBOSS v 6.6.0.0 [71]. The longest similarity index ((similar residues with BLOSUM62 matrix) × 100)/(length of the alignment − total number of gaps in the alignment), was used.

The relationships among members of the hirustasin superfamily and the HMEI family are too complex to be directly observed. We reconstructed phylogenetic trees of the two protein families using IQ-TREE v1.6.12 [72]. The best-fitting models were tested using the ModelFinder module embedded in IQ-TREE. As suggested by the authors of this program, 1000 bootstrap replicates were set to test the support rates of the tree nodes. In addition, to test the potential relationships between the anticoagulant activity and molecular evolution of hirudins, we also collected the sequence and functional information of hirudins from previous reports. We then combined the reported hirudins and those identified in this study and reconstructed the phylogenetic relationships of all sequences using IQ-TREE.

### 2.5. Anticoagulation Analyses of Hirudins

Hirudin was the first identified and most studied antithrombotic protein. The antithrombotic (mainly anticoagulant) activities of hirudin variants from different leech species have been repeatedly analyzed [22]. We used *Pichia pastoris* as expression vector to produce recombinant hirudin variants of *H. manillensis* according to Hu et al. [73] with minor modifications. Briefly, the DNA sequences of hirudin variants were synthesized using chemical synthesis methods and subcloned into pPIC9K-His using EcoRI and NotI restriction enzymes to obtain recombinant plasmids. The recombinant plasmids were linearized with SalI and electroporated into *P. pastoris* strain GS115. The cells were plated on yeast extract peptone-dextrose medium containing 0, 0.25, 0.50, 1.0, and 2.0 mg/mL G418. The positive transformants harboring recombinant hirudin variants were identified through PCR and grown overnight in 5 mL buffered glycerol-complex medium at 30 °C with shaking. After 2 days of culture, hirudin protein expression was induced by daily addition of 1.0% *v*/*v* methanol. The harvested supernatant was then centrifuged, and the protein of interest was extracted with 70% ammonium sulfate.

The antithrombin activity of the recombinant protein was evaluated using the antithrombin titration method as previously described [74], with minor modifications. First, three reagents were prepared: A, 0.5 mg/mL harvested hirudin solution diluted in 0.2 mol/L PBS (phosphate-buffered solution, pH = 7.4); B, 10 mg/mL bovine fibrinogen solution in normal saline; C, thrombin solution with 50 U/mL anticoagulant activity in normal saline. Second, to prepare a 2 mL glass tube, add 100 μL of hirudin solution and 200 μL of reagent B to the tube, and then place the tube in a 37 °C water bath for 5 min. Third, add 5 μL of Reagent C to the tube every 4 min while gently shaking. Fourth, stop adding Reagent C when the liquid starts to solidify and calculate the anticoagulation activity U = (C1 × V1)/(C2 × V2). C1, V1, C2, and V2 indicate the concentration of Reagent C, the total volume of Reagent C added to the tube, the concentration of Reagent A, and the volume of Reagent A used in the reaction, respectively. PBS solution without any proteins was used as control group. For each group, three duplicates were set.

## 3. Results

### 3.1. The Genome Sequencing and Assembling

**Figure 1:**
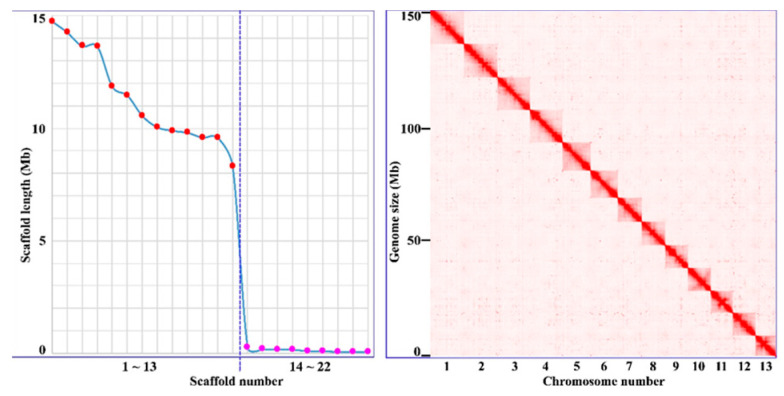
Scaffold and chromosome lengths of *Hirudinaria manillensis* genome.

We used the PacBio HiFi platform for TGS sequencing and performed genome assembly for *H. manillensis*. A total of 23.80 Gb of highly accurate HiFi reads were obtained, with an average length of 18.73 Kb. The de novo assembly generated 34 contigs with a total length of 149.59 Mb and an N50 of 9.58 Mb. We then used the Illumina HiSeq platform for the NGS sequencing of Hi-C libraries, and based on the obtained 21.09 Gb Hi-C reads, we consolidated the contigs into 22 scaffolds with a total length and an N50 of 148.59 Mb and 11.46 Mb, respectively. The first 13 longest scaffolds were 14.76~8.30 Mb in length, while the remaining 9 scaffolds were less than 0.3 Mb each. The highly discontinuous scaffold length distribution and the well-resolved Hi-C maps (Figure 1) clearly represent 13 chromosomes of this species. The 13 chromosomes had a total length of 147.43 Mb, representing 99.22% of the total scaffold length. In addition, we used the Illumina HiSeq platform for NGS sequencing and performed mitochondrial genome assembly using GetOrganelle. A total of 9.52 Gb NGS reads were sequenced, and a circular complete mitochondrial genome of 15,581 bp was obtained. As a result, we obtained a nearly complete genome of *H. manillensis* with a total length of ~150 Mb, including 13 chromosomes, 1 mitochondrial genome, and 9 debris (1.16 Mb). The genome assembly was deposited in the Figshare online repository: https://doi.org/10.6084/m9.figshare.24187377.v1, accessed on 24 September 2023 (Supplementary Materials).

We estimated the completeness of the final assemblies using BUSCO (Benchmarking Universal Single-Copy Orthologs) with the eukaryota_odb10 database. The results showed that 98.0% of the BUSCOs were captured, including 92.5% complete and single-copy BUSCOs, 3.1% complete and duplicated BUSCOs, and 2.4% fragmented BUSCOs. We also used Merqury to estimate the quality of the assembly and obtained a quality score of 33.53. The BUSCO and Merqury results indicated that we had obtained a high-completeness and high-quality genome of this species. We also searched for repeat sequences in the genomes using RepeatModeler and RepeatMasker. A total of 18.75% of the genome was identified as repeats, including retroelements (6.59%), DNA transposons (1.76%), rolling circles (0.20%), unclassified elements (8.41%), satellites (0.2%), and simple repeats (1.8%). No small RNA or low-complexity repeat elements were found. The masked genome was used for gene prediction.

### 3.2. Gene Prediction and Annotation

We used two ab initio prediction approaches (GlimmerHMM and SNAP) and three RNASeq-based prediction approaches (PASA, Stringtie, and BRAKER) to predict protein-coding genes in the masked genomes. The ab initio approaches detected more genes than the RNASeq-based approaches, but with much lower N50 values of the CDS (<1 kb). Of the three RNASeq-based prediction approaches, BRAKER detected the smallest number of genes (25,331) but had the largest N50 values of the CDS (2151 bp) (Table 1). After combination using EVidenceModeler, a total of 21,828 genes were predicted with a CDS N50 of 1731 bp.

To test the prediction sensitivity, we used the well-known hirudin (HV1) as a query to blast the CDS predicted by each approach. The results showed that two hirudin-coding genes were predicted by each of the two ab initio prediction approaches, and three were predicted by the BRAKER approach. Unexpectedly, no such gene was detected by PASA, Stringtie, or the EVidenceModeler integrated sequences (Table 1). Considering all the above factors, we decided to use the CDS and proteins predicted by BRAKER as the relatively best prediction results for further analyses.

We aligned the protein sequences predicted by the BRAKER approach against the NCBI NR, Uniprot TrEMBL, eggNOG, and Pfam databases to perform functional annotations. About 2/3 of the proteins were functionally assigned to the NR, TrEmbl, EggNOG, and Pfam databases, respectively (Table 2). After synthesizing the results of the four assignments, a total of 18,373 (72.53%) genes were functionally assigned or annotated.

### 3.3. Identification of Antithrombotic Genes

By reviewing the literature on leeches, we collected 21 reliable antithrombotic proteins, including 14 coagulation inhibitors, 3 platelet aggregation inhibitors, 3 fibrinolysis enhancers, and 1 tissue penetration enhancer. The anticoagulants can also be further categorized into three groups: (1) thrombin inhibitors, including hirudin and progranulin; (2) Factor Xa inhibitors, including antistasin, lefaxin, and therostasin; and (3) serine protease inhibitors, including hirustasin, guamerin, piguamerin, bdellastasin, poecistasin, eglin, bdellin, leech-derived tryptase inhibitor (LDTI), and *H. manillensis* elastase inhibitor (HMEI) (Table 3). We used the archetypal antithrombotic proteins as queries to blast the gene set predicted by the BRAKER approach. A total of 61 antithrombotic genes were identified, and at least 1 homologous gene was detected for most of the gene families (19 out of 21 gene families, except for therostasin and bdellin). Similarly, we blasted the predicted genes from the previous studies on *H. manillensis* [35,42]. A total of 28 antithrombotic genes from 12 gene families were detected by Guan et al. [42], while 46 antithrombotic genes from 17 gene families were detected by Zheng et al. [35] (Table 4).

To obtain as many antithrombotic genes as possible, we used a so-called BRAKER-plus strategy, which combined manual prediction and BRAKER prediction to improve the prediction of antithrombotic genes. Based on the BRAKER-plus strategy, a total of 72 antithrombotic genes (70 integrated and 2 pseudogenetic) were successfully identified from the 21 gene families, outperforming previous studies. Of the 72 corresponding antithrombotic proteins, 59 were encoded by 12 multi-gene families (hirudin, antistasin, lefaxin, bdellastasin, poecistasin, eglin, HMEI, saratin, apyrase, lumbrokinase, destabilase, and hyaluronidase) that contained 2 or more closely related members. The remaining nine proteins (progranulin, therostasin, hirustasin, guamerin, piguamerin, bdellin, LDTI, GGT, and LCI) were encoded by single gene families. Nine of the twelve multi-gene families were clustered in a single chromosome, while three families (HMEI, lumbrokinase, and hyaluronidase) were involved in two or three chromosomes. The GFF file and predicted CDS of the BRAKER-plus gene prediction approach, and all CDS of the 72 antithrombotic genes were also deposited in the Figshare online repository: https://doi.org/10.6084/m9.figshare.24187377.v1, accessed on 24 September 2023 (Supplementary Materials).

### 3.4. Genetic Variation of Antithrombotic Proteins

**Figure 2:**
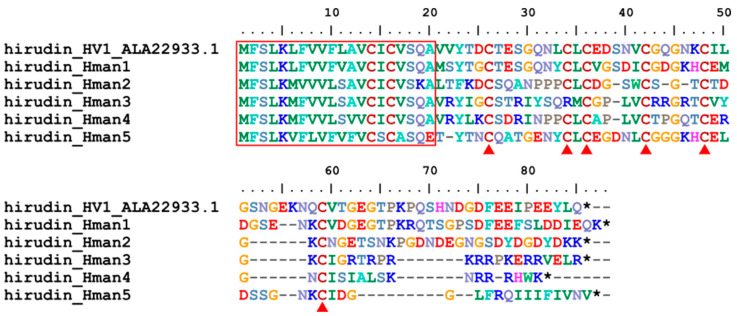
Alignment of the archetypal hirudin (from *H. medicinalis*) and the hirudins identified from the *H. manillensis* genome. Red frame, signal peptide; red triangle, conserved cysteine; *, stop codon (similarly hereinafter).

Hirudin is the first antithrombotic bioactive molecule identified in leeches [79] and has been used for many years as an anticoagulant because it binds directly to thrombin to prevent blood clotting [80]. The first complete amino acid [81] and cDNA [82] sequences of hirudin were determined from *H. medicinalis*. Subsequently, additional subtypes or variants were sporadically reported from *H. medicinalis* [83], *H. manillensis* [84], *Haemadipsa sylvestris* [85], *H. nipponia* [26], and *Whitmania pigra* [86]. However, there has been no genome-wide systematic study of hirudins and their encoding genes in a leech species. Here, five hirudin genes were found in the *H. manillensis* genome. The corresponding five proteins of these genes were 66~85 amino acid residues in length. Online blasting showed that these proteins had a 57.63~77.83% identity with the hirudins deposited in GenBank (Table 5), indicating that these proteins were all hirudin variants. There was a relatively conserved signal peptide region and six cysteine sites (except for hirudin_Hman3 which had a C34R mutation) (Figure 2), which is a typical nature of hirudins to form three disulfide bonds [85]. The sequence similarities between the archetypal hirudin (HV1, ALA22933.1) and those identified in this study were 62.96~78.95%.

**Figure 3:**
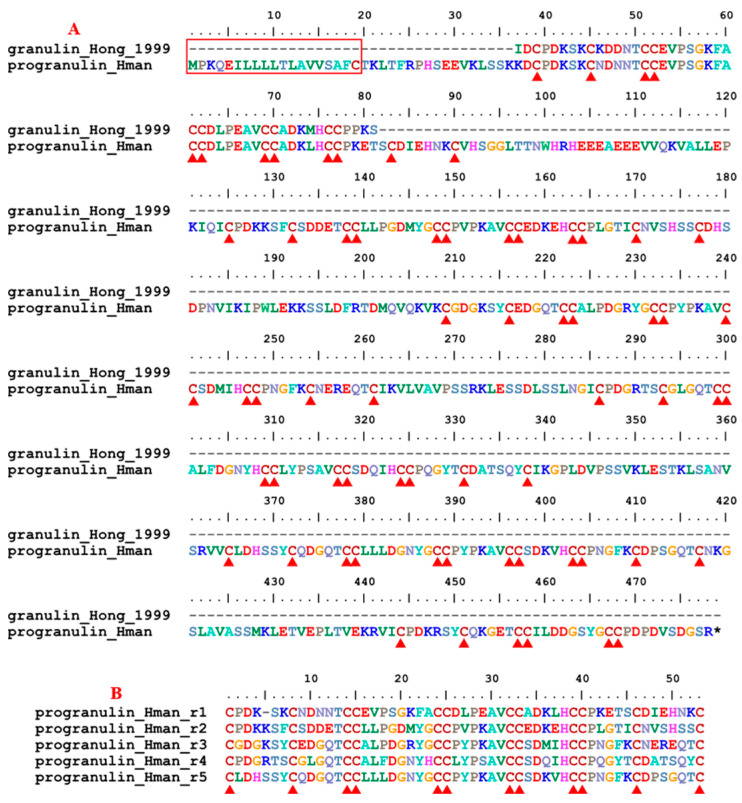
(**A**) Alignment of the archetypal granulin (from *H. nipponia*) and progranulin identified from the *H. manillensis* genome; (**B**) Alignment of the five internal tandem repeats of the *H. manillensis* progranulin.

Granulin is a secreted protein that acts as a key regulator of lysosomal function and as a growth factor involved in inflammation, wound healing, and cell proliferation. The precursor protein, progranulin, consists of a highly conserved tandem repeat of 12 cysteines in the primary sequence [87]. The leech granulin isolated from *H. nipponia* behaved as a thrombin inhibitor [75], although the mechanism was not clear. Here, a single progranulin gene was detected in the *H. manillensis* genome. The corresponding precursor protein contained 478 amino acids including a signal peptide region. The archetypal granulin peptide from *H. nipponia*, which was first shown to have anti-thrombin activities [75], is located in the N-terminus of the proteins. Five conserved internal tandem repeats were found (Figure 3), each containing 12 conserved cysteines. The sequence similarity between the archetypal granulin and *H. manillensis* progranulin was 95.45%.

**Figure 4:**
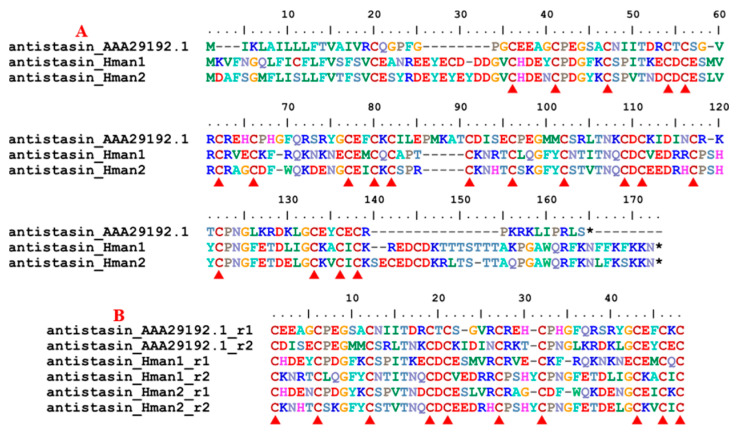
(**A**) Alignment of the archetypal antistasin (from *Haemadipsa officinalis*) and the antistasins identified from the *H. manillensis* genome; (**B**) Alignment of the five internal tandem repeats of the *H. manillensis* antistasin.

Antistasin belongs to a class of serine protease inhibitors characterized by a well-conserved pattern of cysteine residues [88]. Antistasin is a potent anticoagulant that stoichiometrically and selectively inhibits mammalian Factor Xa, a pivotal serine protease in the coagulation cascade [89]. Antistasin has been identified in two *Haementeria* species, *H. officinalis* [90] and *H. ghilianii* (nominated as *ghilanten*) [91], but no homology has been found in other leeches. Here, two antistasin genes were detected in the genome, and the corresponding protein sequences were relatively conserved. Similar to the archetypal antistasin from *Haementeria* [90,92], there were 20 cysteine residues (Figure 4) in all antistasin proteins. In addition, two internal tandem repeats were observed in each of the two antistasin proteins (Figure 4), confirming that these molecules are true antistasins. The sequence similarities between the archetypal antistasin (AAA29192.1) and those identified in this study were 58.33~61.46%.

**Figure 5:**
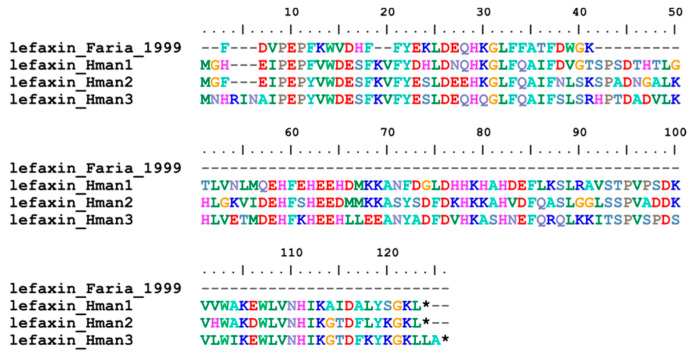
Alignment of the archetypal lefaxin (from *H. depressa*) and the lefaxins identified from the *H. manillensis* genome.

Lefaxin (leech Factor Xa inhibitor), as its name suggests, was also an effective inhibitor of Factor Xa. Lefaxin was first purified from dissected salivary complexes of a leech *H. depressa*. Inhibition of Factor Xa by lefaxin was demonstrated by inhibition of its amidolytic activity and its ability to inhibit thrombin generation in the prothrombinase complex [93]. Three genes have been identified in the *H. manillensis* genome. In contrast to the other Factor Xa inhibitors, no signal peptide or conserved cysteines were found in the corresponding proteins. The archetypal lefaxin from *H. depressa* corresponded to the N-terminus of the six lefaxins (Figure 5). The sequence similarities between the archetypal lefaxin (P86681.1) and those identified in this study were 75.00~78.57%.

**Figure 6:**
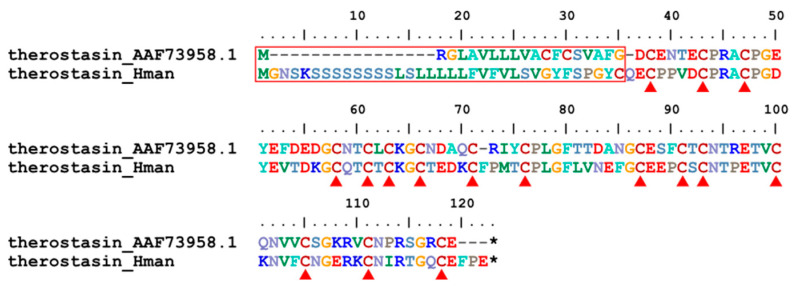
Alignment of the archetypal therostasin (from *T. tessulatum*) and the therostasin identified from *H. manillensis*.

Therostasin is another potent naturally occurring inhibitor of mammalian Factor Xa. It was first isolated from the duck leech *T. tessulatum*. Like the other known inhibitors of Factor Xa, Therostasin is expressed and stored in the cells of the salivary glands of the leech. Therostasin is a cysteine-rich protein with 16 cysteine residues [94]. A therostasin gene has been identified in *H. manillensis*. The corresponding protein sequences contain a signal peptide region that is much longer than that of *T. tessulatum*. In contrast, the functional region was much more conserved between the two leeches, especially the 16 cysteine residues (Figure 6). The sequence similarity between the archetypal therostasin (AAF73958.1) and that of *H. manillensis* was 72.45%.

**Figure 7:**
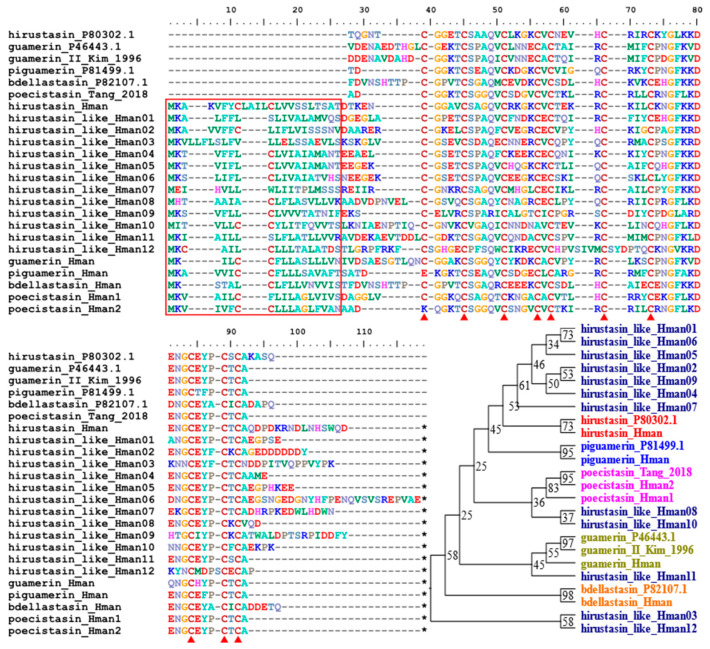
Alignment and phylogenetic relationships of the archetypal hirustasin (from *H. medicinalis*), guamerin (from *H. nipponia*), piguamerin (from *H. nipponia*), bdellastasin (from *H. medicinalis*), and poecistasin (from *H. manillensis*) and their homologous proteins identified from the *H. manillensis* genome in this study.

Hirustasin was a serine protease inhibitor first identified from *H. medicinalis* [95]. Unlike antistasin or therostasin, hirustasin neither inhibited in vitro blood coagulation nor the amidolytic activity of isolated mammalian Factor Xa. In contrast, hirustasin inhibited trypsin, chymotrypsin, tissue kallikrein, and neutrophil cathepsin G [95], which have activating effects on Factor X and enhanced activity on Factor XII [11]. Later, additional analogues of hirustasin were identified from other leeches: guamerin and piguamerin from *H. nipponia* [96,97], guamerin variant II from *Whitmania edentula* [98], bdellastasin from *H. medicinalis* [99], and poecistasin from *H. manillensis* [76]. Due to the high similarity in both sequence and function, we referred to these proteins as hirustasin superfamily members. In this study, a total of 18 hirustasin superfamily members were detected. We combined all these members and the archetypal hirustasin (P80302.1), guamerin (P46443. 1) as well as guamerin II [98], piguamerin (P81499.1), bdellastasin (P82107.1), and poecistasin [76], and after alignment, we attempted to determine their affiliation through a phylogenetic analysis using IQ-TREE. The results showed that one hirustasin, one guamerin, one piguamerin, one bdellastasin, and two poecistasin were identified. The remaining 12 proteins were not clustered to hirustasin and its analogues; hence, they were nominated as hirustasin-like proteins for convenience. All proteins had a signal peptide and 10 conserved cysteines (Figure 7). The sequence similarities between the archetypal hirustasin (P80302.1) and those identified in the *H. manillensis* genome were 55.32~78.72%.

**Figure 8:**
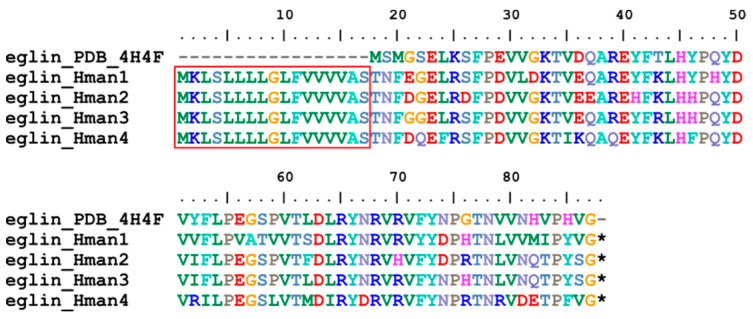
Alignment of the archetypal eglin (from *H. medicinalis*) and the eglin identified from the *H. manillensis* genome.

Eglin (elastase-cathepsin G leech inhibitor) is a small protein with potent inhibitory activity against chymotrypsin and subtilisin-like serine proteinases acting on non-cationic substrates [100]. This molecule was first identified from *H. medicinails* and is able to inhibit platelet activation induced by cathepsin G [101]. Two eglin variants (eglin B and eglin C) were detected from a single species [102], suggesting that they are encoded by a multi-gene family. Four eglin genes were identified in the genome of *H. manillensis*, and the corresponding proteins were relatively conserved (Figure 8). The signal peptide region was detected, but as previously reported [102], no cysteine residues were found in the proteins. The sequence similarities between the archetypal eglin (PDB accession: 4H4F) and those identified in this study were 83.08~88.41%.

**Figure 9:**
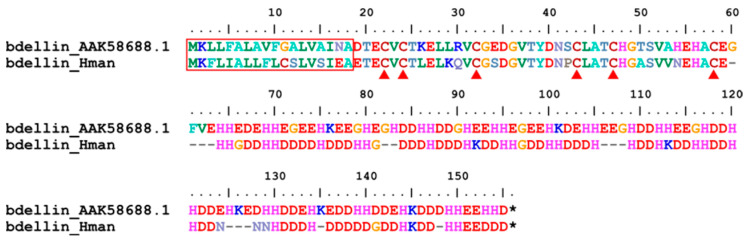
Alignment of the archetypal bdellin (from *H. medicinalis*) and the bdellin identified from the *H. manillensis* genome.

Bdellin is an inhibitor of trypsin, plasmin, and sperm acrosin. It was first discovered in 1969 [103], and its protein sequence was first identified from *H. medicinalis* [104]. It has Kazal serine proteinase inhibitors with a typical domain of six cysteine residues forming a 1–5, 2–4, 3–6 disulfide bond pattern [105]. A bdellin gene was detected in the *H. manillensis* genome. The corresponding protein sequences were relatively conserved, including a signal peptide region of 19 amino acids, an N-terminal region of about 40 amino acids and six cysteines, and a C-terminal region with a highly charged repetitive sequence (Figure 9). The sequence similarity between the archetypal bdellin (AAK58688.1) and that of *H. manillensis* was 78.17%.

**Figure 10:**
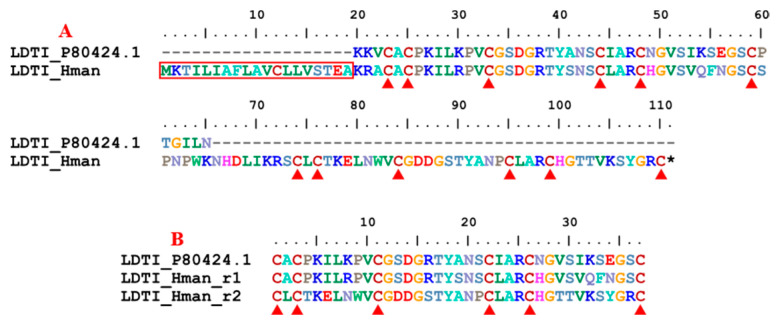
(**A**) Alignment of the archetypal LDTI (from *H. medicinalis*) and the LDTI identified from the *H. manillensis* genome; (**B**) Alignment of the archetypal LDTI and two internal tandem repeats of the *H. manillensis* LDTI.

Leech-derived tryptase inhibitor (LDTI) is another Kazal-type serine protease inhibitor that inhibits mast cell tryptase in particular, but also trypsin and chymotrypsin [106]. LDTI was first isolated from *H. medicinalis* [107]. Recombinant LDTI was shown to inhibit thrombin and trypsin, thereby prolonging blood clotting time [108,109]. Here, we identified a single LDTI gene in the *H. manillensis* genome. There was a signal peptide region in the corresponding proteins. The rest of the functional region was also relatively conserved, especially on the 12 cysteines (Figure 10). Sequence alignment showed that the archetypal LDTI from *H. medicinalis* matched the N-terminal of the functional regions of the LDTI from *H. manillensis*. Further analysis revealed that, similar to antistasin mentioned above, two internal tandem repeats of six cysteines are in these proteins (Figure 10). The sequence similarity between the archetypal LDTI (P80424.1) and that from *H. manillensis* was 92.50%.

**Figure 11:**
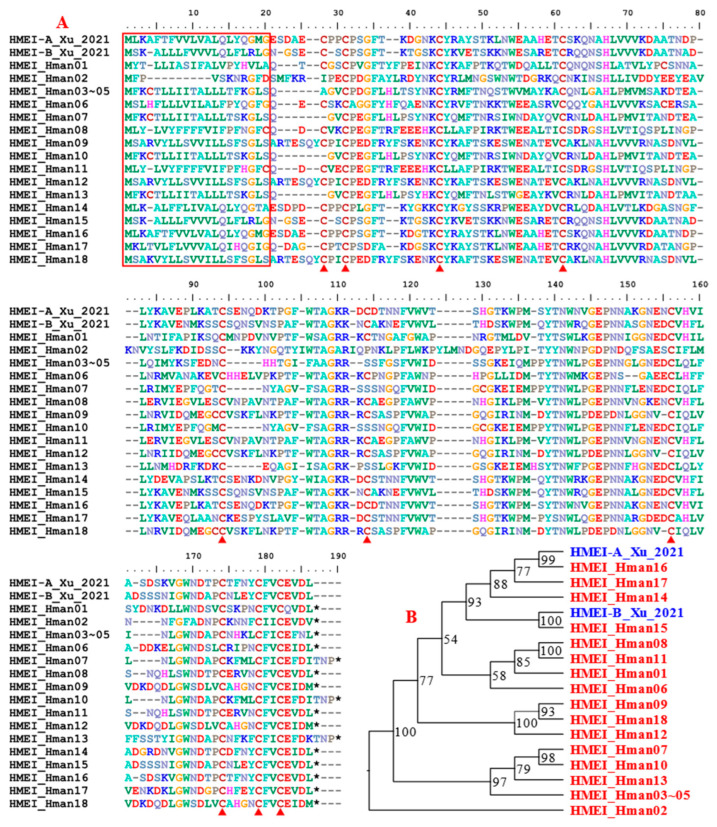
Alignment (**A**) and phylogenetic relationships (**B**) of the archetypal HMEI-A and HMEI-B (from *H. manillensis*) and the HMEIs identified from the *H. manillensis* genome in this study.

HMEI (*H. manillensis* elastase inhibitor) is a newly discovered elastase inhibitor from *H. manillensis*. The protein belongs to a multigene family because at least two members, HMEI-A and HMEI-B, have been detected. The HMEI-A showed potent abilities to inhibit elastase and as a result inhibited the formation of neutrophil extracellular trap (NET). Although the authors did not test the activity of HMEI-B, a similar function was expected based on the high-sequence identity, especially the highly conserved cysteine residues [77]. Since NET plays an important role in abnormal thrombus formation [110], its inhibition by HMEI proteins will lead to additional antithrombus outcomes. A total of 18 HMEI genes were detected in the *H. manillensis* genome, indicating that they belong to a large gene family. Of the 18 corresponding proteins, three (HMEI_Hman03 ~ HMEI_Hman05) were identical to each other. Phylogenetic analysis showed that HMEI_Hman16 and HMEI_Hman15 were closely related to HMEI-A and HMEI-B, respectively (Figure 11). The proteins were highly variable in both sequence length and amino acid sites. The proteins had a signal peptide and ten cysteines were clearly visible. The pairwise similarity between the archetypal HMEI-A [77] and the newly detected proteins was 52.83~99.41%.

**Figure 12:**
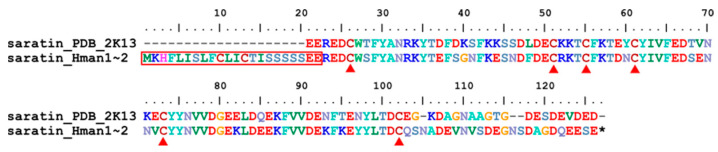
Alignment of the archetypal saratin (from *H. officinalis*) and the saratins identified from the *H. manillensis* genome.

Saratin was an inhibitor of collagen-platelet interaction [111,112]. By competitively inhibiting the binding of von Willebrand factor (vWF) to collagen [113], the protein showed anti-platelet aggregation activity and helped prevent blood clotting [114]. Saratin was first isolated from *H. medicinails* [115], but another protein called leech antiplatelet protein (LAPP) which was found almost simultaneously from the leech *H. officinalis* [116], was suggested to be functionally homologous to saratin [30]. Recently, an additional homology was found in *H. nipponia* [114]. Two identical saratin genes were found in the *H. manillensis* genome. Similar to hirudins, the saratins had a conserved signal peptide region and six cysteine sites (Figure 12). The sequence similarity between the archetypal saratin (PDB: 2K13) and those identified in this study was 84.69%.

**Figure 13:**
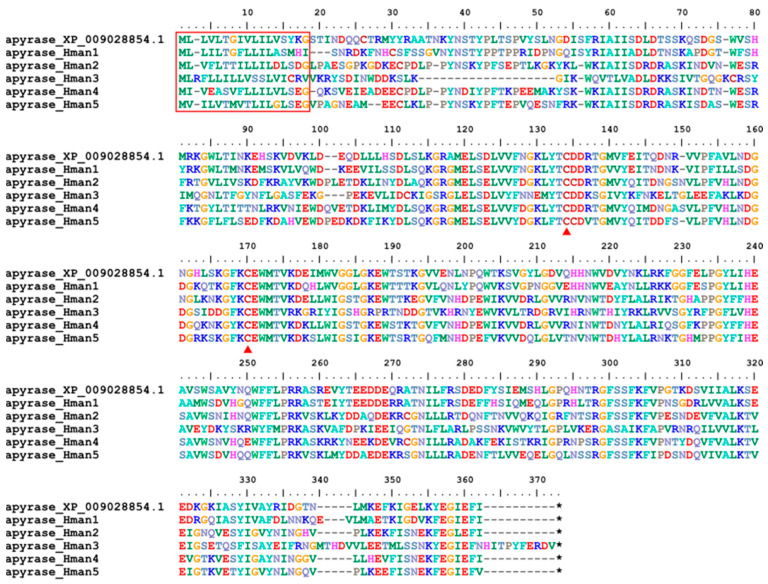
Alignment of the archetypal apyrase (from *H. robusta*) and the apyrases identified from the *H. manillensis* genome.

Apyrase (adenosine 5′-diphosphate diphosphohydrolase) is a nonspecific inhibitor of platelet aggregation by acting on adenosine 5′-diphosphate, arachidonic acid, platelet-activating factor, and epinephrine. Apyrase activity is thought to be responsible for the inhibition of ADP-induced platelet aggregation, as indicated by the apparent release of apyrase from salivary glands into the host during blood feeding [117]. The bioactivities of apyrase have been studied using salivary extract from the Nile leech *Limnatis nilotica* [118], but sequence information was only available for a non-hemophagous leech (*H. robusta*, XP_009028854.1). Here, we identified five apyrase proteins from the *H. manillensis* genome. One signal peptide region and two conserved cysteine sites were detected (Figure 13). The sequence similarities between the *H. robusta* apyrase (XP_009028854.1) and the six newly obtained proteins were 58.64~81.71%.

**Figure 14:**
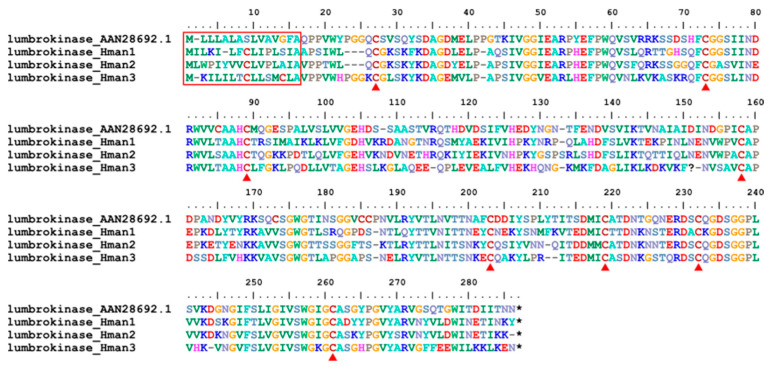
Alignment of the archetypal lumbrokinase (from *L. rubellus*) and the lumbrokinases identified from the *H. manillensis* genome.

Lumbrokinase is a fibrinolytic serine protease enzyme with potent fibrinolytic activity and inhibitory effects on platelet aggregation [119]. Lumbrokinase was first extracted from the earthworm *L. rubellus* [120] and later from other earthworm species such as *Eisenia fetida* [121]. Here, we report for the first-time lumbrokinase from leeches. Three forms of lumbrokinase were detected in the *H. manillensis* genome. These proteins had high sequence similarity to the archetypal lumbrokinase from *L. rubellus* (GenBank No. AAN28692.1). All leech lumbrokinases and the archetypal lumbrokinase from *L. rubellus* were relatively conserved in the signal peptide region and 14 cysteine residues (Figure 14), suggesting that these proteins also play important roles in platelet aggregation. The sequence similarities between the archetypal lumbrokinase (AAN28692.1) and those from *H. manillensis* were 67.68~69.20%.

**Figure 15:**
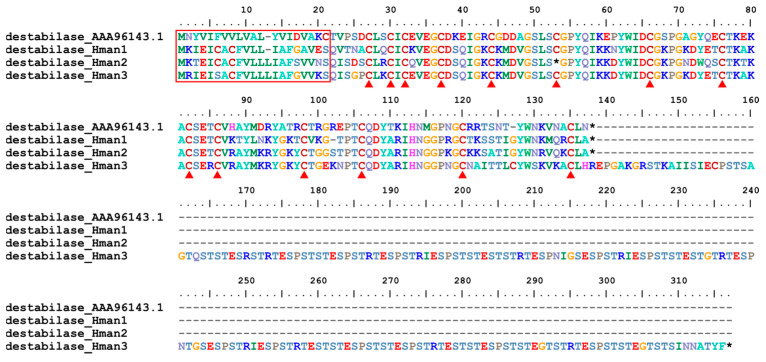
Alignment of the archetypal destabilase (from *H. medicinalis*) and the destabilases identified from the *H. manillensis* genome.

Destabilase is an endo-e(c-Glu)-Lys isopeptidase that cleaves isopeptide bonds formed by transglutaminase (Factor XIIIa) between glutamine c-carboxamide and the e-amino group of lysine. The molecule can disrupt covalent bonds formed between fibrin monomers under the influence of Factor XIIIa in blood plasma [122]. Destabilase was first detected in the salivary secretions of *H. medicinalis* [123], and three forms have been identified [124]. Similar to *H. medicinalis*, three forms were also detected in the *H. manillensis* genome. The corresponding protein sequences were relatively conserved, with the same signal region length and 14 cysteine residues (Figure 15). Surprisingly, the C-terminus of destabilase_Hman3 was extremely elongated, making its total length more than twice that of the other forms. The elongated sequences had many irregular short repeats such as “ESP” and “EST” (Figure 15). The sequence similarities between the archetypal destabilase (AAA96143.1) and those identified in this study were 70.08~73.60%.

**Figure 16:**
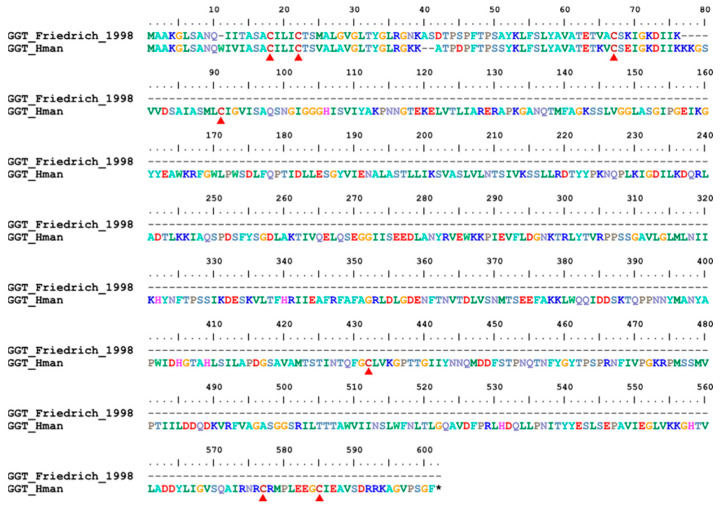
Alignment of the archetypal GGT (from *H. medicinalis*) and the GGT identified from the *H. manillensis* genome.

γ-glutamyl transpeptidase (GGT) is a cell surface enzyme that hydrolyzes the γ-glutamyl bond of extracellular reduced and oxidized glutathione, initiating its cleavage into glutamate, cysteine (cystine), and glycine. It is a critical enzyme in maintaining cellular redox homeostasis and is used as a marker of liver disease and cancer [125]. A homology of mammalian GGT was isolated from the salivary gland secretion of *H. medicinalis*. This leech GGT was observed to have proteolytic activities on factor XIIIa cross-linked fibrin [78]. A GGT gene was found in the genome of *H. manillensis*. Six cysteines but no signal peptide region was detected in this protein (Figure 16). The sequence similarity between the archetypal GGT [78] and those identified in this study was 91.78%.

**Figure 17:**
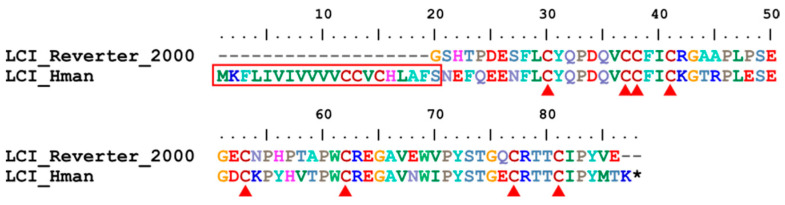
Alignment of the archetypal LCI (from *H. medicinalis*) and the LCI identified from the *H. manillensis* genome.

Leech carboxypeptidase inhibitor (LCI) is a metallo-carboxypeptidase inhibitor isolated from the medicinal leech *H. medicinalis*. LCI binds tightly to pancreatic carboxypeptidases A1, A2, B and to plasma carboxypeptidases B. Assuming that leeches secrete LCI during feeding, the inhibitor could help maintain the fluid state of blood by inhibiting thrombin-activatable fibrinolysis inhibitors [126]. Here, a single LCI gene was detected in the *H. manillensis* genome. The corresponding protein had a signal peptide region and eight cysteines (Figure 17), which are critical for the structure and functional integrity of this protein [127]. After alignment, the archetypal LCI of *H. medicinalis* perfectly matched the C-terminal of the LCI of *H. manillensis*. The sequence similarity between the archetypal LCI [28] and that of *H. manillensis* was 86.89%.

**Figure 18:**
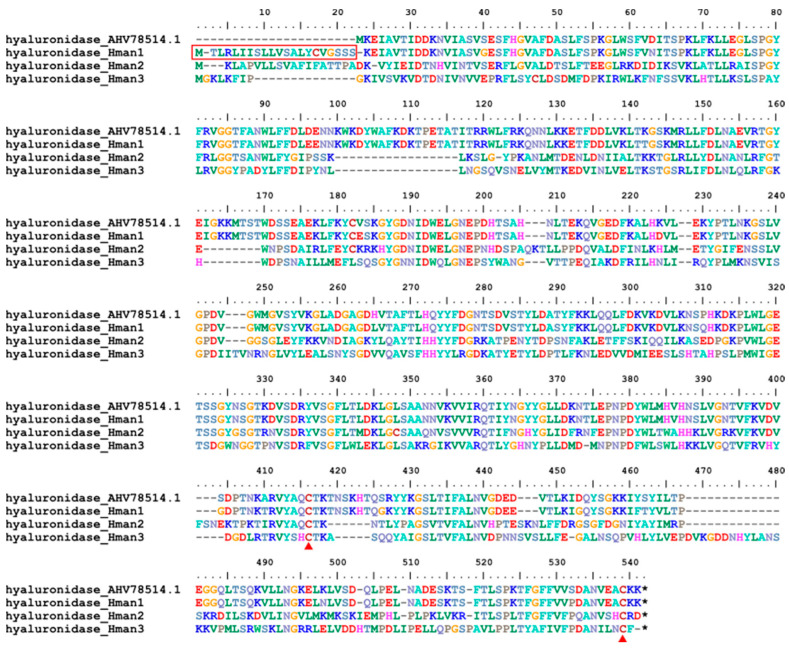
Alignment of the archetypal hyaluronidase (from *H. nipponia*) and the hyaluronidases identified from the *H. manillensis* genome.

Hyaluronidase is a spreading or diffusing substance that modifies the permeability of connective tissue through the hydrolysis of endoglucoronidic bonds of hyaluronic acid [128]. Leech hyaluronidase, first isolated from *H. medicinalis*, was the most specific enzyme known for the identification of hyaluronic acid [129]. After the bite, leeches immediately release hyaluronidase to facilitate tissue penetration and spread of their bioactive molecules [130]. It has been suggested that there are three types of leech hyaluronidase [131], although sequence information is scarce. Here, for the first time, we report all three hyaluronidase genes of *H. manillensis*. One corresponding protein (hyaluronidase_Hman1), but not the other two, had a signal peptide region. Two conserved cysteines were detected in these proteins (Figure 18). The similarities between the archetypal hyaluronidase (from *H. nipponia*, AHV78514.1) and the newly obtained six proteins were 62.39~97.55%.

### 3.5. Anticoagulation of Recombinant Hirudins

Hirudin is the first identified and most concerned antithrombotic protein. Here, we use *P. pastoris* as a eukaryotic expression vector to produce recombinant hirudins. The results showed that hirudin_Hman1, hirudin_Hman2, and hirudin_Hman5 exhibited obvious anticoagulant activities with mean ± SD of 960.1 ± 142.4, 508.8 ± 12.4, and 803.0 ± 101.3 U/mg, respectively. In contrast, hirudin_Hman3 and hirudin_Hman4 and the control group showed no anticoagulant activity.

**Figure 19:**
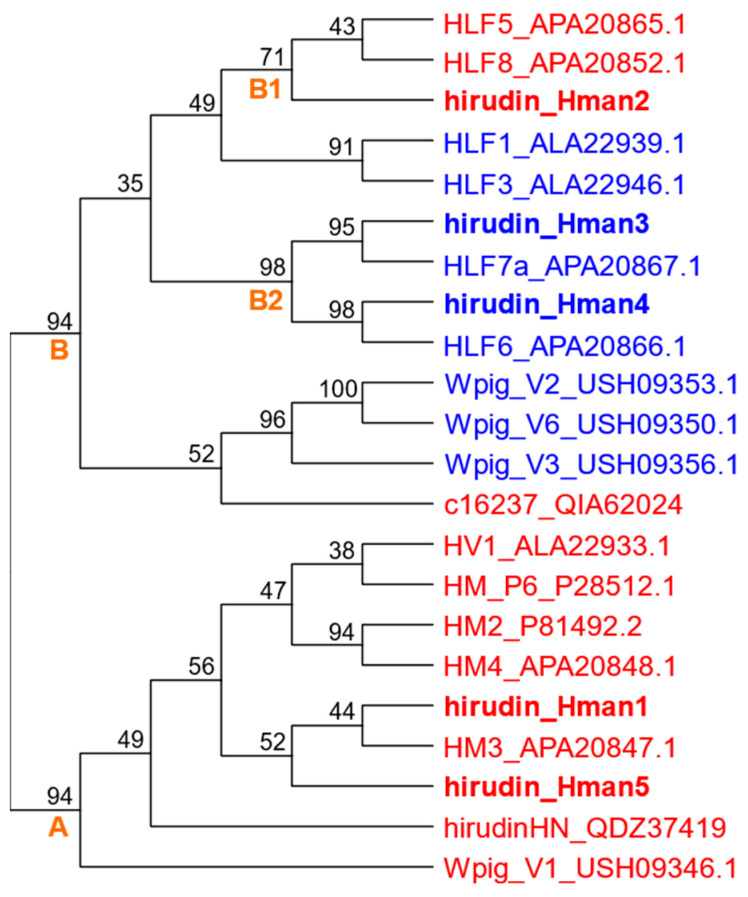
Phylogenetic relationship of the five hirudins hirudin_Hman1 ~ hirudin_Hman5 identified from this study and all available protein sequences whose anticoagulant activity had been measured (red, active proteins; blue, inactive proteins; A, clade in which all hirudins are active proteins; B, clade containing both active and inactive hirudins; B1, subclade in which all hirudins are active proteins; B2, subclade in which all hirudins are inactive proteins).

We collected all available protein sequences whose anticoagulant activity had been tested and combined with the five hirudins identified in this study and reconstructed a phylogenetic tree using IQ-TREE. The results showed that the proteins with and without anticoagulant activity clustered into distinct clades. In particular, active hirudin_Hman1 and hirudin_Hman5 were distributed in a monophyletic clade (clade A), which included seven active hirudins from *H. medicinalis* (HV1), *H. manillensis* (HM_P6, HM2, HM3, and HM4), *H. nipponia* (hirudinHN), and *W. pigra* (Wpig_V1). The active hirudin_Hman2 was distributed in another monograph (group B1), which included two active hirudins from *H. manillensis* (HLF5 and HLF8). In contrast, the inactive hirudin_Hman3 and hirudin_Hman4 were distributed in another monograph (group B2) with inactive hirudins from *H. manillensis* (HLF7a and HLF6) (Figure 19).

## 4. Discussion

A high-quality genome is essential for effective identification and analysis of functional proteins. Recent high-throughput sequencing and assembly technologies make it easier to obtain a chromosome-scale genome. Here, we used state-of-the-art third-generation sequencing (PacBio HiFi) and next-generation sequencing (Illumina Hi-C, Survey and RNASeq) to obtain a nearly complete chromosome-scale genome of *H. manillensis*. A total of 13 chromosomes were identified, consistent with the recent study [35]. However, the chromosomes in our study account for 99.22% of the total length of the scaffolds, which is much higher than that in the recent study (95.92% in [35]). The BUSCO analyses showed that 95.6% of complete BUSCOs were captured. In contrast, after reanalyzing the previously published genomes [35], only 88.7% complete BUSCOs were captured for the *H. manillensis* genome. The Merqury analyses yielded a quality score of 33.5, which is also higher than that of Zheng et al. [35] (32.1). In addition, we assembled a complete circular mitochondrial genome for this species. As a result, based on the above parameters, it is almost certain that we have obtained the most integrated genome for *H. manillensis* to date.

In contrast to the high quality of the genome assemblies, the results of the genetic prediction of antithrombotic genes were not very satisfactory. To test the prediction quality of different methods, we used the finally identified hirudins as query sequences to BLAST (tblastn) the CDS data sets predicted using these methods. Among the five hirudins, 2~3 hirudins were successfully predicted by GlimmerHMM, SNAP, and Braker, while none were predicted by PASA or Stringtie. Unexpectedly, no hirudin gene was found in the consensus prediction results generated by EVidenceModeler. It should be noted that the prediction of antithrombotic genes (at least for hirudins) does not necessarily seem to be better with higher completeness of genomes than in previous studies [35,42,86], indicating that formalistic prediction by program pipelines is not sufficient to identify antithrombotic genes.

Using a so-called BRAKER-plus strategy, combining BRAKER prediction and manual prediction, we finally identified 72 genes from 21 gene families involved in antithrombotic functions. All protein products of these genes had more than 50% sequence similarity to their corresponding archetypal proteins, indicating that our identification methods are reliable. Except for lefaxin, eglin, apyrase, and hyaluronidase, most of the antithrombotic proteins were cysteine-rich, forming three or more disulfide bonds. By increasing protein stability, disulfide bonds were thought to make protein structures relatively independent of their amino acid sequences, thus acting as buffers against deleterious mutations and allowing for accelerated sequence evolution [132]. Interestingly, three of the cysteine-rich antithrombotic protein families (LDTI, antistasin, and granulin) have internal tandem repeats containing 6, 10, and 12 conserved cysteines, respectively. The pattern of internal tandem oligopeptide repeats is not uncommon in natural proteins; however, the functional and evolutionary significance of such a pattern has not been well studied [133].

Leeches have been used as a medical and pharmaceutical resource for many centuries, massive bioactive proteins have been reported, and many species (*Hirudo* spp., *Hirudinaria* spp., *Haemadipsa* spp., *Haementeria* spp., *Theromyzon* spp., etc.) have been involved in related studies [134]. The high variation of antithrombotic proteins and their coding genes cause confusion in the research and application of these substances. Taking hirudin as an example, in addition to the archetypal hirudin identified from *H. medicinalis*, several alternative names have been used: hirudin-like factors that have no anticoagulant activity [83], haemadin from *Haemadipsa* spp. [85], and bufrudin from *Hirudinaria* spp. [135]. In addition, most of the previous studies involve only a single gene of a gene family. In fact, many proteins are encoded by multigene families. In the precent study, the high-quality genomes and careful personalized analysis provide an opportunity to systematically investigate the antithrombotic proteins as well as genes of *H. manillensis*.

With respect to the three antithrombotic drugs (anticoagulation, antiplatelet aggregation, and fibrinolysis), we broadly classified the antithrombotic proteins of leeches in this study into three categories: inhibitors of coagulation, inhibitors of platelet aggregation, and enhancers of fibrinolysis. Of the 21 antithrombotic protein families identified, 14 (67%) were involved in coagulation inhibition, including 2 thrombin inhibitors (hirudin and granulin), 3 Factor Xa inhibitors (antistasin, therostasin, and lefaxin), and 9 serine protease inhibitors (hirustasin, guamerin, piguamerin, bdellastasin, poecistasin, eglin, bdellin, LDTI, and HMEI). Three gene families (saratin, apyrase, and lumbrokinase) were involved in the inhibition of platelet aggregation. Another three (destabilase, GGT, and LCI) were involved in enhancing fibrinolysis. The remaining family, hyaluronidase, does not belong to these three categories, but since it facilitates tissue penetration and diffusion for the other 20 protein families, its indirect antithrombotic function is not negligible.

Hirudin is the most potent thrombin-specific inhibitor identified to date and is a representative pharmacologically active substance in leeches [25,136]. In the pharmacopoeia of the People’s Republic of China (PPRC), antithrombin activity is the key index for determining the quality of leeches [74]. The anticoagulation analysis of *H. manillensis* hirudins in this study showed that three (hirudin_Hman1, hirudin_Hman2, and hirudin_Hman5) out of five hirudins had anticoagulant activity. We also collected the sequence and functional information of hirudins from previous reports and, then, tested the phylogenetic relationships of the reported hirudins and those from this study. The results showed that the proteins with and without anticoagulant activity clustered into separate clades. For example, the active hirudin_Hman1, hirudin_Hman2, and hirudin_Hman5 had closer relationships with proteins that were confirmed to have anticoagulant activity. In contrast, the inactive hirudin_Hman3 and hirudin_Hman4 were more phylogenetically related to proteins confirmed to have no anticoagulant activity. These results repeatedly confirmed that at least three functionally active hirudins were simultaneously distributed in a single *H. manillensis* genome. These findings, together with the presence of two pseudogenetic genes (*lumbrokinase_Hman3* and *destabilase_Hman2*), remind us that some other antithrombotic proteins may also suffer from loss of biological activity. The actual biological function of the other 67 proteins will need to be validated by laboratory experiments in the future.

In conclusion, in this study, we provided an almost complete, high-quality genome of *H. manillensis*. Combined with automatic and manual prediction, we identified 72 antithrombotic genes involving 21 gene families. The functions of the corresponding proteins include anticoagulation, anti-platelet aggregation, fibrinolysis, and drug diffusion. We also provided the complete CDS and protein sequences and their variation information for all 72 antithrombotic genes/proteins. This is the most comprehensive collection of genomes and leech antithrombotic biomacromolecules to date. Our results will greatly facilitate the research and application of leech derivatives for medical and pharmaceutical purposes of thrombosis.

## Figures and Tables

**Table 1:** Statistics of predicted protein-coding genes in *H. manillensis* genome.

Annotation Approach	Gene Number	Total CDS Length (Mb)	N50 of CDS (bp)	No. of Hirudins
GlimmerHMM	56,061	31,047,090	873	2
SNAP	45,826	26,341,403	762	2
PASA	37,111	33,145,509	1122	0
Stringtie	32,919	40,238,799	1554	0
BRAKER	25,331	36,676,815	2151	3
EVidenceModeler	21,828	26,863,541	1731	0

**Table 2:** Functional annotation of proteins predicted from the *H. manillensis* genome.

Annotation Approach	Gene Number	Percentage
NR	17,411	68.73%
TrEmbl	17,421	68.77%
EggNOG	15,952	62.97%
Pfam	15,675	61.88%
integration	18,373	72.53%

**Table 3:** Archetypal sequence of antithrombotic proteins from leeches (note: as an exception, *Lumbricus rubellus* is an earthworm).

No.	Protein	Leech Species	Accession/Reference	Function
1	hirudin	*Hirudo medicinalis*	ALA22933.1	coagulation inhibitor
2	granulin	*Hirudo nipponia*	[75]	coagulation inhibitor
3	antistasin	*Haementeria officinalis*	AAA29192.1	coagulation inhibitor
4	lefaxin	*Haementeria depressa*	P86681.1	coagulation inhibitor
5	therostasin	*Theromyzon tessulatum*	AAF73958.1	coagulation inhibitor
6	hirustasin	*H. medicinalis*	P80302.1	coagulation inhibitor
7	guamerin	*H. nipponia*	P46443.1	coagulation inhibitor
8	piguamerin	*H. nipponia*	P81499.1	coagulation inhibitor
9	bdellastasin	*H. medicinalis*	P82107.1	coagulation inhibitor
10	poecistasin	*H. manillensis*	[76]	coagulation inhibitor
11	eglin	*H. medicinails*	PDB: 4H4F	coagulation inhibitor
12	bdellin	*H. nipponia*	AAK58688.1	coagulation inhibitor
13	LDTI	*H. medicinails*	P80424.1	coagulation inhibitor
14	HMEI	*H. manillensis*	[77]	coagulation inhibitor
15	saratin	*H. officinalis*	PDB: 2K13	platelet aggregation inhibitor
16	apyrase	*Helobdella robusta*	XP_009028854.1	platelet aggregation inhibitor
17	lumbrokinase	*L. rubellus*	AAN28692.1	platelet aggregation inhibitor
18	destabilase	*H. medicinails*	AAA96143.1	fibrinolysis enhancer
19	GGT	*H. medicinails*	[78]	fibrinolysis enhancer
20	LCI	*H. medicinails*	[28]	fibrinolysis enhancer
21	hyaluronidase	*H. nipponia*	AHV78514.1	tissue penetration enhancer

**Table 4:** Identified antithrombotic genes from *H. manillensis* genome in previous studies and in this study.

No.	Gene Family	Guan et al. [42]	Zheng et al. [35]	BRAKER Prediction	BRAKER-Plus Prediction
1	*hirudin*	1	3	3	5
2	*progranulin*	1	1	1	1
3	*antistasin*	0	2	2	2
4	*lefaxin*	3	3	3	3
5	*therostasin*	0	1	0	1
6	*hirustasin/hirustasin-like*	1/3	1/5	1/11	1/12
7	*guamerin*	0	0	1	1
8	*piguamerin*	0	0	1	1
9	*bdellastasin*	0	1	1	1
10	*poecistasin*	0	1	0	2
11	*eglin*	0	3	3	4
12	*bdellin*	0	0	0	1
13	*LDTI*	1	1	1	1
14	*HMEI*	4	9	15	18
15	*saratin*	1	1	2	2
16	*apyrase*	5	5	5	5
17	*lumbrokinase*	3	3	3	3
18	*destabilase*	0	1	3	3
19	*GGT*	1	1	1	1
20	*LCI*	1	0	1	1
21	*hyaluronidase*	3	3	3	3
—	total	28	46	61	72

**Table 5:** NCBI online blast results of the five hirudins predicted in this study.

Hirudin	Target Species	Protein	Accession	Identity
hirudin_Hman1	*H. manillensis*	HM1	Q07558.1	76.83%
hirudin_Hman2	*H. manillensis*	HLF8	APA20852.1	57.63%
hirudin_Hman3	*H. manillensis*	HLF7	APA20868.1	62.96%
hirudin_Hman4	*H. manillensis*	HLF6	APA20866.1	73.68%
hirudin_Hman5	*H. manillensis*	HM1	Q07558.1	68.33%

## Data Availability

The sequence data (clean reads) of PacBio, Hi-C, Survey, and RNASeq were deposited in GenBank under Project PRJNA1019887.

## Supplementary Materials

The following supporting information can be downloaded at the Figshare online repository: https://doi.org/10.6084/m9.figshare.24187377.v1 (accessed on 24 September 2023). These are the genome assembly (1Hman.genome.fa), GFF file of BRAKER-plus gene prediction approach (2Hman.genome.gff), all predicted CDS (3Hman.cds.fa), and all CDS of 72 antithrombotic genes (4Hman.antithrombotic.cds.fa) of *H. manillensis*.
